# Inflammation- and Tissue Remodeling-Related Gene Responses in Skeletal Muscle of Heart Failure Patients Following High-Intensity Interval Training

**DOI:** 10.31083/j.rcm2402046

**Published:** 2023-02-06

**Authors:** Andrea Tryfonos, Georgios Tzanis, Εleftherios Karatzanos, Michael Koutsilieris, Serafim Nanas, Anastassios Philippou

**Affiliations:** ^1^Department of Life Science, European University Cyprus, 2404 Nicosia, Cyprus; ^2^Department of Physiology, Medical School, National and Kapodistrian University of Athens, 11527 Athens, Greece; ^3^Clinical Ergospirometry, Exercise & Rehabilitation Laboratory, Evaggelismos Hospital, National and Kapodistrian University of Athens, 11527 Athens, Greece

**Keywords:** heart failure, cardiac rehabilitation, inflammation, skeletal muscle remodelling

## Abstract

**Background:**

Peripheral myopathy consists a hallmark of heart failure (HF) and has been associated with poor prognosis. Inflammation has been suggested to dominate this pathology, while exercise training is typically associated with the induction of anti-inflammatory mechanisms. However, the current knowledge regarding the involvement of inflammation-related genes in the exercise training-induced muscle adaptations in HF patients is very limited. Given that high-intensity interval training (HIIT) alone or combined with strength training (COM) has gained ground in HF cardiac rehabilitation, this study aimed to investigate the local muscle expression of inflammatory and tissue remodeling factors in HF patients, who underwent 3 months of these training schemes. In addition, we examined whether these exercise training-induced gene expression responses are associated with changes in exercise capacity in those patients.

**Methods:**

Thirteen male patients with chronic HF (age: 51 ± 13 y; body mass index (BMI): 27 ± 4 kg/$m^{2}$) were randomly assigned to a 3-month exercise program consisted of either HIIT (N = 6) or COM training (N = 7). Muscle tissue biopsies were obtained from vastus lateralis pre- and post-training and transcriptional changes in interleukin 6 (*IL-6*), interleukin 8 (*IL-8*), tumor necrosis factor-1 alpha (*TNF-1α*), urokinase-type plasminogen activator (*uPA*), urokinase-type plasminogen activator receptor (*uPAR*), and transforming growth factor-beta 1 (*TGF-β1*) were quantified by RT-PCR.

**Results:**

An overall increase in the expression levels of selected inflammatory (*IL-8, TNF-1α*) and remodeling factors (*uPAR*) was found post-training (*p *< 0.05), while *IL-6*, *uPA* and *TGF-β1* gene expression remained unchanged (*p *> 0.05). The observed alterations did not differ between training groups. Additionally, *IL-8 *changes were found to be correlated with the improvement in exercise capacity post-training (*p *< 0.05).

**Conclusions:**

This is the first study demonstrating an increase in intramuscular inflammatory and remodeling key factors induced by HIIT or COM training in HF patients. Combining these observations with our previous findings of improved muscle hypertrophy and capillarization post-training in these patients, the findings of the present study may suggest that inflammatory responses are part of an ongoing remodeling process in the exercising skeletal muscle.

**Clinical Trial Registration:**

NCT02387411.

## 1. Introduction

Whilst heart failure (HF) is predominantly a disorder of central haemodynamics [1], it has been demonstrated to induce deteriorating effects on multiple peripheral tissues including skeletal muscle [2]. Indeed, there is growing evidence suggesting that HF leads to structural and functional alternations within skeletal muscle resulting in muscle cachexia [2, 3], which has been known as ‘*the muscle hypothesis*’ of chronic HF [4, 5]. Indeed, Fülster *et al*. [6] reported that one in five HF patients experienced muscle wasting, including those with reduced and preserved (≥40%) left ventricular ejection fraction (LVEF), implying that muscle cachexia may be the most frequent co-morbidity among these patients. Importantly, patients with muscle wasting were more likely to exhibit reduced LVEF, exercise capacity, and muscle strength, all of which contribute to poor prognosis in HF [6].

Systemic and local inflammatory responses appear to be involved in early but also advanced stages of this pathology [7]. Specifically, studies have shown an inverse correlation between the level of inflammatory cytokines, tumor necrosis factor (TNF)-1α and interleukin (IL)-6, in the circulation and the clinical severity of the disease [8, 9], including exercise intolerance and reduced skeletal muscle mass in HF patients [10]. Recently, a thorough examination of skeletal muscle biopsies in HF patients and age-matched controls performed by Bekfani *et al*. [11] has pointed that HF patients with lower muscular endurance presented with elevated expression of the inflammatory biomarker GDF-15 and atrophy-related factors [Atrogin1, F-box only protein 32 (FBXO-32), and Myostatin-2 (MSTN-2)], which further supports the detrimental effect of inflammation towards muscle wasting. However, the role of inflammation in skeletal muscle appears to be more complex; studies have associated pro-inflammatory markers with muscle wasting [10], while others supported the crucial role of inflammation in tissue remodeling processes, including muscle hypertrophy and angiogenesis [12, 13, 14, 15, 16, 17].

Inflammation has been identified as the mechanism by which exercise training-induced skeletal muscle repair and hypertrophy is initiated [12, 18, 19]. Briefly, following muscle-damaging exercise muscle fibers secrete pro-inflammatory factors that recruit immune cells to scavenge muscular debris, allowing muscle regeneration and tissue remodeling [13, 18, 19]. A crucial balance between pro-inflammatory and anti-inflammatory factors appears to attenuate an excessive inflammatory reaction and interactive cytokine responses promote the progression or resolution of muscle inflammation [14]. In HF patients, although there is wealth of evidence demonstrating a reduction of circulating cytokines following exercise training [20, 21], however only one study has measured the levels of intramuscular pro-inflammatory markers following a 6-month exercise training program [22]. In that study a decrease in pro-inflammatory gene expression in the exercised muscles has been reported post-training, along with the absence of monocytes/macrophages infiltration in them [22], suggesting a possible adaptive downregulation of the local inflammatory program as part of the tissue remodeling process.

Interestingly, exercise characteristics, such as type and intensity, as well as the duration of exercise training, is likely to trigger different inflammatory responses [23]. For instance, strenuous and/or resistance exercise have been associated with greater muscular adaptations predominantly due to a larger extent of exercise-induced muscle damage [23]. Notably, superior muscular and cardiopulmonary adaptations following high intensity interval training (HIIT) and/or strength training compared to traditional aerobic exercise of moderate intensity has also been documented in patients with HF [24, 25, 26, 27]. Thus, according to the most recent guidelines of European Society of Cardiology-ECS (2020) [28] and American Heart Association-AHA (2017) [29], combined strength and aerobic training protocols have now been graded as a *Class I* recommendation in the treatment of HF patients. Nevertheless, there is lack of information regarding the intramuscular responses of inflammatory and tissue remodeling factors potentially as part of the local adaptive mechanisms induced by such exercise protocols in patients with HF.

Our group has previously found an overall increase in exercise capacity, muscle hypertrophy and capillarization in patients with HF that followed 3 months of either a HIIT or a combined HIIT with strength training program [24, 30]. Based on that evidence, the present study investigated further whether intramuscular inflammation and tissue remodeling factors contribute to those adaptations, advancing our understanding on the molecular pathways that mediate beneficial effects of exercise training on skeletal myopathy in HF patients. Specifically, we investigated and compared the effects of the same HIIT versus combined HIIT with strength training (COM) programs [24, 30] on the transcriptional changes in inflammation- and tissue remodeling-associated factors in skeletal muscle of stable HF patients. In addition, this study examined the potential associations between those gene expression responses and changes in exercise capacity in these patients.

## 2. Methods

### 2.1 Subjects

Thirteen male patients with stable HF [age 51 ± 13 years; body mass index (BMI) 27 ± 4 kg/$m^{2}$, LVEF 37 ± 9%], New York Heart Association (NYHA) functional class ≤ III, at optimal medical treatment, consented to participate in this study. Detailed information about the inclusion and exclusion criteria for the participants of this study are given elsewhere [24]. The study was approved by the Human Study Committee of our institution.

Specific components of the present study have been previously published regarding the effects of exercise training on muscle hypertrophy [24] and angiogenesis [30] in the HF patients. The current work aimed to examine the gene expression of inflammation- and tissue remodeling-related factors in skeletal muscle of these patients in response to exercise training, in order to further characterize the molecular effects of exercise-based cardiac rehabilitation on HF-induced skeletal myopathy. The baseline characteristics of the patients aa well as their medications are shown in Table 1 (Ref. [24, 30]).

**Table 1.** **Patients’ baseline characteristics and medications used [24, 30]**.

		HIIT (N = 6)	COM (N = 7)
Age (years)		47 ± 13 (31–61)	53 ± 12 (33–68)
BMI (kg/$m^{2}$)		27 ± 6 (19.3–35.6)	27 ± 2 (24.6–31.1)
HF etiology (ICM/non-ICM)		2/4	3/5
NYHA (I/II/III)		2/3/1	1/5/1
Weber class (A/B)		3/3	3/4
${\mathrm{VO}}_{2}$peak (mL/kg/min)		21.1 ± 4.7 (16.4–26.9)	20.0 ± 4.8 (16.0–29.0)
LVESD (mm)		43 ± 12 (28–65)	47 ± 13 (29–63)
LVEDD (mm)		59 ± 7 (51–71)	62 ± 9 (45–75)
LVEF (%)		37 ± 10 (18–45)	38 ± 8 (27–50)
PCWP (mmHg)		9 ± 6 (2–16)	11 ± 8 (3–25)
mPAP (mmHg)		19 ± 10 (11–33)	21 ± 7 (12–29)
RAP (mmHg)		2 ± 2 (0–4)	4 ± 3 (0–9)
Hemoglobin (g/dL)		14.5 ± 2.4 (10.8–16.6)	13.9 ± 1.0 (12.6–15.5)
CI (L/min/$m^{2}$)		2.2 ± 0.6 (1.4–2.9)	2.3 ± 0.4 (1.6–2.8)
Medications (%)			
	Amiodarone	50	29
	β-Blockers	100	100
	Diuretics	67	71
	ACE inhibitors/ARB	100	100
	MRAs	100	100

Values are given as mean ± SD (lowest-highest). BMI, body mass index; HF, heart failure; ICM, ischemic cardiomyopathy; NYHA, New York Heart Association functional class; ${\mathrm{VO}}_{2}$peak, peak oxygen consumption; LVESD, left ventricular end-systolic diameter; LVEDD, left ventricular end-diastolic diameter; LVEF, left ventricular ejection fraction; PCWP, pulmonary capillary wedge pressure; mPAP, mean pulmonary artery pressure; RAP, right atrial pressure; CI, cardiac index; ACE, angiotensin-converting enzyme; ARB, angiotensin receptor blockers; MRAs, Aldosterone receptor antagonists.

### 2.2 Study Design 

Detailed description of the design and methods of this study as well as of the exercise training protocols used are given elsewhere [24]. All participants underwent either the HIIT (N = 6) or the combined HIIT with strength (COM, N = 7) exercise training for 3 months, 3 sessions per week. Any missed sessions were compensated at the end of the 12-week scheduled program, so that each patient completed 36 exercise sessions. Both training protocols were of the same total duration (31 minutes). Muscle tissue biopsies were collected from the patients before and after the 3-month training program and transcriptional changes in inflammation- and tissue remodeling-related genes were examined.

### 2.3 Cardiopulmonary Exercise Testing

Subjects performed an 8–12 min ramp-incremental exercise test, using a gas exchange analyser (Quark PFT, Cosmed, Rome, Italy) as previously descripted [24]. Briefly, patients requested to perform incremental leg exercise, while oxygen uptake (${\mathrm{VO}}_{2}$), carbon dioxide output (${\mathrm{VCO}}_{2}$), and ventilation (VE) were measured breath by breath at rest, throughout exercise, and for 5 mins during recovery. The gas exchange measurements served to calculate ${\mathrm{VO}}_{2}$peak (mL • ${\mathrm{kg}}^{-1}$ • $\min^{-1}$). For this work, ${\mathrm{VO}}_{2}$peak (mL • ${\mathrm{kg}}^{-1}$ • $\min^{-1}$) percentage change pre-to-post exercise training has been used to examine its possible association with the fold changes in the mRNA expression of inflammation and remodeling-related factors that examined.

### 2.4 Skeletal Muscle Biopsies

At the beginning and 48–72 h after the last exercise training session of the program, percutaneous needle biopsies of the vastus lateralis muscle were obtained using the Bergstrom technique [31], snap frozen and stored, as previously described [24].

### 2.5 RNA Extraction and Semiquantitative Reverse Transcription–Polymerase Chain Reaction Analysis

RNA extraction from muscle tissue samples and semiquantitative real-time polymerase chain reaction (RT-PCR) were performed to identify differences between mRNA expression before and after the 3-month training program. These procedures and the real-time PCR parameters used are described in detail elsewhere [24]. The primer set sequences used for the specific detection of each gene are given in Table 2. Specifically, primers were designed against *IL-6, IL-8, TNF-1α*, transforming growth factor-beta 1 (*TGF-β1*), urokinase-type plasminogen activator (*uPA*) and its receptor (*uPAR*), while glyceraldehyde 3-phosphate dehydrogenase (*GAPDH*) was applied as housekeeping gene (internal standard). The specificity of the primers for the corresponding gene was confirmed by the melting curve and the electrophoretic analyses of the RT-PCR products.

**Table 2.** **The sequence of the specific sets of primers used in RT-PCR analyses**.

Target gene	PCR primer sequence	Product size (bp)
*IL-6*	F: 5′-CCTGACCCAACCACAAATGC-3′	157
	R: 5′-ATCTGAGGTGCCCATGCTAC-3′	
*IL-8*	F: 5′-CCACCGGAAGGAACCATCTC-3′	279
	R: 5′-TTCCTTGGGGTCCAGACAGA-3′	
*TNF-1α*	F: 5′-AAGAGTTCCCCAGGGACCTCT-3′	229
	R: 5′-ACATGGAGTAGATGAGGGT-3′	
*TGF-β1*	F: 5′-CTACTACGCCAAGGAGGTCAC-3′	236
	R: 5′-ATGGAGTCGTTGGCCACGA-3′	
*uPA*	F: 5′-GTCTACCTGGGTCGCTCAAG-3′	374
	R: 5′-CAGTGGTGGTTTTACGACAC-3′	
*uPAR*	F: 5′-CATGCAGTGTAAGACCAACGGGGA-3′	253
	R: 5′-TGAGACCGGCCCGACAGTGGTAT-3′	
*GAPDH*	F: 5′-CATCACTGCCACCCAGAAGA-3′	438
	R: 5′-TCCACCACCCTGTTGCTGTA-3′	

F, Forward primer; R, Reverse primer.

### 2.6 Statistics

Changes in the mRNA expression were assessed using two-way repeated-measures ANOVA. Where significant F ratios were found for main effects or interaction (*p *< 0.05), pairwise comparisons were performed using Fisher’s least significant difference (LSD) test. Paired *t*-tests (pre-post) in each exercise group were used to examine the effects within each group. The Pearson correlation coefficient (r) was used to examine potential associations between factors examined. Results are presented as mean ± SD and significance was set at *p ≤* 0.05. Statistical Package for the Social Sciences (SPSS V. 26, IBM Corp., Chicago, IL, USA) was used for all analyses.

## 3. Results

### 3.1 Expression of Inflammation- and Tissue Remodeling-Related Factors

**Fig. 1.**
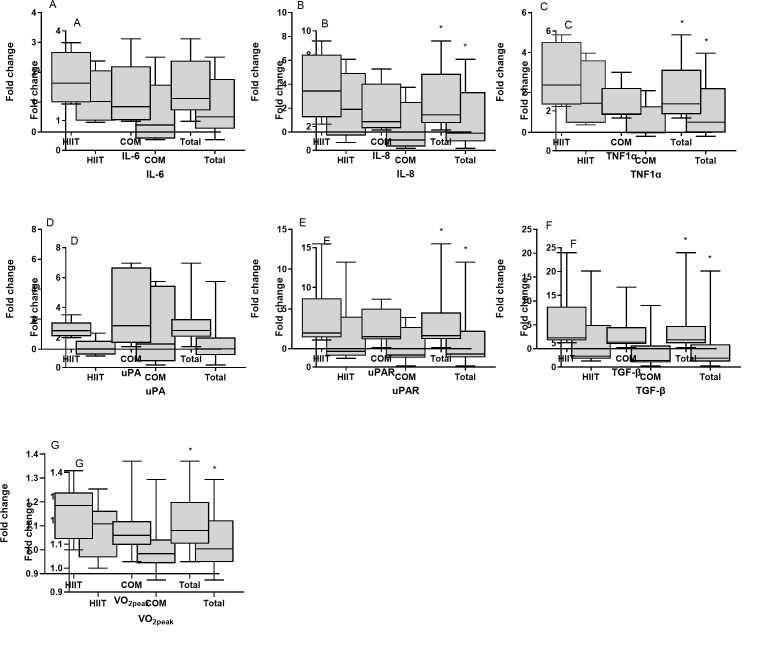
**Expression of inflammation- and tissue remodeling-related factors after exercise training**. The box-plot diagrams represent the fold changes in the mRNA expression of (A) interleukin 6 (*IL-6*), (B) interleukin 8 (*IL-8*), (C) tumor necrosis factor-1 alpha (*TNF-1α*), (D) urokinase plasminogen activator (*uPA*), (E) urokinase plasminogen activator receptor (*uPAR*), (F) transforming growth factor-beta (*TGF-β1*) in the trained skeletal muscle, (G) as well as the fold changes of oxygen consumption (${\mathrm{VO}}_{2}$peak) in HF patients following either high-intensity-interval training (HIIT; N = 6), or combined HIIT with strength exercise training (COM; N = 7), as well as in the total number of patients (N = 13). * Significantly different compared to pre-exercise levels *p *< 0.05.

Compared with the pre-exercise training values, the expression of *IL-8* was significantly increased (*p* = 0.019), while these increases were not different between the training groups (*p* = 0.167). In addition, after the completion of the exercise training program there was an overall increase of the *TNF-1α* (*p* = 0.010) and *uPAR* (*p* = 0.046) mRNA expression compared to baseline levels, again without these changes being significant between the groups (*p* = 0.073 and *p* = 0.455, respectively). Moreover, an upward trend was revealed in the expression of *IL-6* (*p* = 0.083), *TGF-β1* (*p* = 0.067) and *uPA* (*p* = 0.126) after the exercise training, however these increases did not reach statistical significance (*p *> 0.05). Similarly, no differences between the exercise groups were observed regarding *IL-6* (*p* = 0.321), *TGF-β1* (*p* = 0.507) and *uPAR* (*p* = 0.356) expression responses post-training. Moreover, paired t-tests were performed separately within HIIT and COM group revealing that except for *TNF-1α*, which was significantly increased following HIIT (*p* = 0.005), no pre-post differences (*p *> 0.05) were revealed following either HIIT or COM. Fig. 1 shows the fold changes in the transcriptional levels of the inflammation- and tissue remodeling factors pre-to-post exercise training in the training groups aa well as in the total number of patients.

### 3.2 Associations between Inflammatory/Tissue Remodeling Genes Expression and Exercise Capacity 

**Fig. 2.**
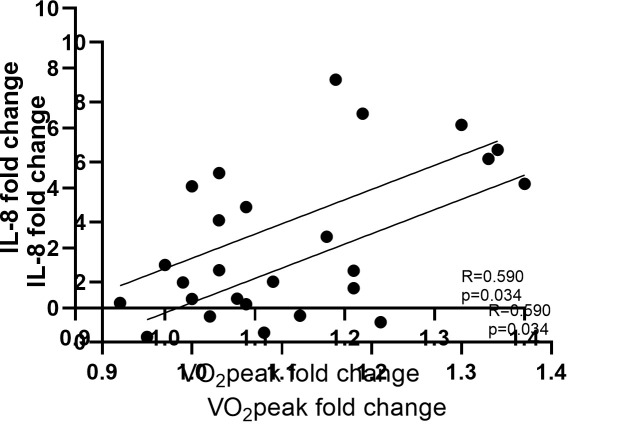
**Association between the fold changes of interleukin 8 *(IL-8*) in the trained skeletal muscle and the changes in exercise capacity expressed as fold change of peak oxygen consumption (${\mathrm{VO}}_{2}$peak) in the total number of HF patients (N = 13)**.

Given that exercise capacity may possess a prognostic value for HF, we examined the potential associations between the exercise training-induced fold changes in the expression of the genes examined and the fold changes in exercise capacity [${\mathrm{VO}}_{2}$peak (mL • ${\mathrm{kg}}^{-1}$ • $\min^{-1}$)] after the completion of the 3-month exercise training. Detailed information regarding the alterations of exercise capacity following HIIT and COM protocol, as well as in the total number of patients of both training groups are given in our previous work [24]. For this work, fold (percentage) change of ${\mathrm{VO}}_{2}$peak was used, which exhibited an overall increase following exercise training (*p* = 0.002), while this increase did not reach significance between the training groups (*p* = 0.176; Fig. 1G). Moreover, exercise capacity changes post-training were found to be positively correlated with the fold changes of *IL-8* expression (R = 0.590, *p* = 0.034; Fig. 2). *IL-6* (R = 0.161, *p* = 0.600), *TNF-1α* (R = 0.307, *p* = 0.307), *TGF-β1* (R = 0.290, *p* = 0.336),* uPA* (R = –0.038, *p* = 0.902), and *uPAR* (R = 0.357, *p* = 0.232) expression responses were not associated with the changes of exercise capacity after exercise training.

## 4. Discussion

Skeletal myopathy occurred in HF patients is strongly associated with poor prognosis [6]. Inflammation has been suggested to dominate this pathology [7], while exercise training is typically proposed to counteract muscle cachexia modulating muscle inflammatory status [32, 33]. Nevertheless, there is a growing body of evidence documenting a beneficial role of exercise-induced intramuscular inflammation, which may be functionally related to muscle repair and growth adaptations [12, 13]. With regard to HF patients, however, most of the existing data have associated exercise training with systemic inflammatory profile [32, 33, 34] and only one study has directly measured local, intramuscular inflammation status following exercise training, in which patients requested to perform a traditional aerobic exercise on a daily basis [22]. Given the wealth of studies demonstrating the superior effects of HIIT or combined HIIT with strength training compared to traditional aerobic protocols in HF patients [24, 27, 35, 36, 37], the presented study was the first that investigated and compared the intramuscular expression of inflammatory and tissue remodeling factors following these specific exercise training schemes.

The main findings of our study were that 3 months of either HIIT or combined HIIT with strength training program resulted in similar increases in the expression of pro-inflammatory and tissue remodeling genes in skeletal muscles of HF patients. In addition, fold changes in the intramuscular *IL-8* expression were positively correlated with the improvement of exercise capacity in these patients, potentially implying a beneficial role of exercise training-induced local inflammation, as part of an on-going remodeling process in the exercising muscles. Combining these observations with the induction of skeletal muscle hypertrophy [24] and angiogenesis [30] that we have previously reported in these particular groups of HF patients following the same exercise training protocols, we might assume that an intramuscular inflammation program may be associated with muscle remodeling and adaptations to exercise training.

Exercise-induced unaccustomed mechanical loading of skeletal muscle can stimulate the activation of aseptic inflammation, the local production of cytokines and the extracellular matrix (ECM) remodeling program [13], which include pro- and anti-inflammatory factors, and factors of the *TGF-β* family [14]. Skeletal muscle is one of the few adult mammalian tissues that can undergo robust remodeling and its regeneration following mechanical overloading is one of the proposed mechanisms by which exercise training leads to skeletal muscle tissue repair and hypertrophy [12, 18, 19]. Specifically, local muscle inflammation appears to orchestrate muscle repair and growth by activating tissue remodeling, hypertrophic and angiogenetic pathways [13, 14]. Muscle cells and a variety of other cells secrete cytokines, including *TNF-1α* [38], *IL-6* [39], and *IL-8 * [40], and other factors to contribute to specific aspects of inflammation and facilitate tissue repair/regeneration, angiogenesis, and hypertrophy [13, 14].

The present study showed a similar increase in pro-inflammatory cytokines (*IL-8, TNF-1α*) in skeletal muscles of HF patients following 3 months of either HIIT or combined HIIT with strength exercise training, while the local expression of *IL-6* remained unchanged after either exercise training program. These findings are in contrast with those of a previous study reporting a reduction in local gene expression *of IL-6 *and *TNF-1α* in HF patients [22]. However, the differences between the two studies regarding the exercise stimuli, i.e., 20 minutes casual aerobic training daily vs. HIIT or combined HIIT with strength training, and the duration of the overall training (6 months vs. 3 months), may somehow explain the discrepancy between these studies. Specifically, HIIT alone or combined with muscle strengthening exercise is likely to induce greater adaptations to skeletal muscle given its more powerful stimuli compared with aerobic exercise, while this might trigger a greater local inflammatory response. Moreover, anti-inflammatory effects of exercise training usually require a longer duration than the 3-month training period of the present study [41]. Furthermore, intramuscular expression of inflammatory cytokines, including *TNF-1α, IL-6*, and *IL-8* remained unchanged following 8 weeks [42] or increased after 10 weeks [43] of similar exercise programs, i.e., HIIT, endurance or strength training. Importantly, those studies were conducted in clinical populations, i.e., patients with chronic obstructive pulmonary disease (COPD) and rheumatoid arthritis, characterized by muscle cachexia, while interestingly, resistance exercise has been shown to result in muscle fiber hypertrophy, despite the intramuscular increase of TNF-1α in patients with type II diabetes [44]. In addition, other studies indicated an overall improvement in the prognosis of patients with chronic heart failure (CHF) following exercise training or other interventions (heart resynchronization, including increased exercise capacity and muscle capillary density without reductions in systemic [45] or local muscle inflammation [46]. Overall, the findings of those studies may indicate a functional relation of intramuscular inflammation to muscle remodeling and growth adaptations following exercise training in patients with CHF, questioning local inflammation as an index of the clinical severity of the disease.

The findings of the present study further support a potential functional role of local inflammation in the exercise-induced muscle adaptations and clinical benefits, revealing that intramuscular increase of *IL-8* following the 3-month cardiac rehabilitation was positively associated with the improvements in exercise capacity of these HF patients. Specifically, *IL-8* has been found to act as a local messenger to promote angiogenesis via its receptor [47] and given our recent findings documenting elevated capillary density and increased angiogenetic activity in these patients [30], we might speculate that exercise training-induced *IL-8* increase may be involved in the angiogenic/remodeling program of the exercised muscles, supporting the improvement of functional capacity of these patients. Indeed, increased muscular capillarization facilitates oxygen transport [48], which in return contributes to the improvement exercise tolerance. Although there are, yet, no studies showing a causative association of local *IL-8* expression to exercise capacity, a recent study observed a positive relation between circulating *IL-8* levels and improved performance in marathon runners [49]. Further studies are required to corroborate the potential beneficial role of intramuscular *IL-8* expression in exercise training-induced adaptations particularly in patients with CHF.

Local muscle inflammation can activate tissue remodeling pathways and a continuous coordination between inflammatory and remodeling factors is required for an efficient structural remodeling and adaptation of skeletal muscle [50]. Specifically, components of the *uPA/uPAR/TGF-β* bioregulation system have been implicated as key modulators of skeletal and cardiac muscle regeneration, since they contribute to ECM degradation and reconstitution and, thus, to muscle tissue remodeling [50, 51]. Specifically, *TGF-β*, which exists in at least five isoforms, namely, *TGF-β1–5*, is an important cytokine that regulates the homeostasis of multiple biological processes including cell growth and motility, apoptosis, and ECM synthesis [14, 15]. During the course of inflammation, *TGF-β* initially acts as a pro-inflammatory factor and later promotes resolution of inflammation [14]. The activation of *uPA/uPAR* system enables *TGF-β1* to promote extracellular matrix remodeling while an excessive, long-term expression of *TGF-β1* has been associated with muscle fibrosis [50]. In the present study, we found an overall increase of *uPAR* expression, though without significant changes in *uPA* and *TGF-β1* expression in skeletal muscle of HF patients following 3 months of either HIIT or combined HIIT with strength training. It is generally known that *uPAR* affects angiogenesis and muscle hypertrophy via the interaction with its ligand *uPA * [52, 53]. The increased expression of *uPAR* without the concurrent increase of *uPA* may suggest a differential or sequential time course of expression of these two factors during a long-term remodeling program of skeletal muscle tissue. Moreover, *uPAR* may also bind to other ligands e.g., vitronectin, and also interact with other receptors (e.g., VEGFR-2) [54], implying alternative actions of this receptor, independently of *uPA* binding. More studies are required to further investigate the expression pattern of the *uPA/uPAR/TGF-β* system components during exercise training and reveal their potential role(s) in training-induced muscle adaptations.

Overall, to the authors’ knowledge this is the first study that examined and compared the expression changes of key inflammatory and tissue remodeling factors in skeletal muscle after a HIIT or combined HIIT with resistance training program in patients with CHF, revealing a gene expression profile compatible with exercise-induced cellular and functional adaptations of the trained muscles that we previously had characterized in the same CHF patients [24, 30].

## 5. Limitations

The potential of this study to detect significant differences between groups regarding the expression of the factors explored may be limited by its small sample size. Moreover, the ability to confirm the functional significance of the expression changes in the inflammation- and tissue remodeling-related factors at the protein level is limited by the transcriptional nature of the study and, thus, future studies are needed to evaluate this question. Overall, further studies are required to reach definite conclusions regarding the impact of the type of exercise-training, HIIT or HIIT in combination with strength training, on the induction of inflammatory and tissue remodeling factors in skeletal muscle, not only after the completion but also at various time points during the training programs in patients with CHF. It’s worth mentioning that an age-matched group (N = 13), which did not participate in any training program nor undergo muscle biopsies, had been also included in this study as a control group in comparison with the effects of both exercise training programs (HIIT and combined HIIT) as a whole on aerobic capacity [24]. Retrospectively, we think that this control group would have been utilized to compare the inflammatory/remodeling responses of skeletal muscle in those patients with the responses observed in the training groups of the study. Thus, further studies that will include a control group not participating in an exercise-based rehabilitation program are encouraged, to examine whether CHF patients exhibit an exaggerated inflammatory response to exercise, given their skeletal myopathy. Finally, our observations on exercise-induced inflammation have been limited to skeletal muscle and we did not collect information regarding systemic inflammation status. As such, further studies should also include parallel measurements of inflammatory factors in the circulation to characterize the overall inflammatory response to exercise training programs in CHF patients.

## 6. Conclusions

This study provided insights regarding the intramuscular molecular responses of key inflammatory and tissue remodeling factors to different exercise training programs, within the context of characterizing a potential network of biological processes that regulate the exercise-induced adaptive alterations of skeletal muscle in patients with CHF. These processes might ultimately counteract skeletal myopathy and improve exercise capacity of those patients. We found an overall upregulation of pro-inflammatory and tissue remodeling factors following both HIIT and combined HIIT with strength training program, which may suggest that inflammatory responses are part of an ongoing remodeling process in the exercising muscle. Given that these types of exercise training have gained significant ground in cardiac rehabilitation of patients with CHF, more studies are required to further describe the molecular signature of skeletal muscle adaptive remodeling during and after different exercise training programs in these patients typically characterized by muscle wasting.

## Data Availability

The datasets used and/or analyzed during the current study are available from the corresponding author on reasonable request.
